# Exploring the effects of wearing facemasks on stair safety characteristics in young adults

**DOI:** 10.1371/journal.pone.0324333

**Published:** 2025-05-22

**Authors:** Timmion K. Skervin, Toby J. Ellmers, Elmar C. Kal, William R. Young, Rebecca L. Walker, Emily Wharton, Neil M. Thomas, Constantinos N. Maganaris, Mark A. Hollands, Richard J. Foster

**Affiliations:** 1 Faculty of Science, Research to Improve Stair Climbing Safety (RISCS), School of Sport and Exercise Sciences, Liverpool John Moores University, Liverpool, United Kingdom; 2 School of Science and Technology, Nottingham Trent University, Nottingham, United Kingdom; 3 Department of Brain Sciences, Imperial College London, London, United Kingdom; 4 Centre for Cognitive and Clinical Neuroscience, Department of Health Sciences, College of Health, Medicine and Life Sciences, Brunel University of London, Uxbridge, United Kingdom; 5 Department of Public Health and Sports Sciences, St Luke’s Campus, University of Exeter, Exeter, United Kingdom; 6 Pupil Labs GmbH, Berlin, Germany; Drexel University School of Biomedical Engineering Science and Health Systems, UNITED STATES OF AMERICA

## Abstract

**Introduction:**

Facemasks are worn in many industries to protect from infections and harmful substances. Asian countries historically have a wide adoption of facemasks; though due to the COVID-19 pandemic, facemask wearing is also common in western countries. The lower visual field provides important information for safe stair negotiation. A loose fit facemask may obstruct the lower visual field and negatively affect stair negotiation. Pinching a facemask nose clip provides contour around the nose which may reduce lower visual occlusion and negative stair behaviour effects. Here, we explored the effect of wearing a Type IIR facemask and nose clip pinch adjustment on lower visual field occlusion and stair walking behaviour

**Method:**

Eight young adults ascended and descended stairs with; 1) no facemask, 2) unadjusted facemask, 3) customised facemask (nose clip pinched). Measurements included peak head flexion, lower visual field occlusion, stair duration, foot clearance, foot placement, margins of stability, Conscious Movement Processing and anxiety.

**Results:**

Unadjusted increased lower visual occlusion during descent (unadjusted = 32° ± 14° vs no facemask = 11° ± 14°, p < 0.001), (unadjusted vs customised = 21° ± 15°, p = 0.009) and ascent (unadjusted = 47° ± 12° vs no facemask = 25° ± 11°, p < 0.001), (unadjusted vs customised = 35° ± 11°, p = 0.005). Unadjusted increased conscious movement processing during descent (unadjusted = 16 ± 5 vs no face mask 11 ± 4, p = 0.040) and ascent (unadjusted = 16 ± 5 vs no face mask = 10 ± 3, p = 0.044). Bayesian inference indicated moderate evidence for the alternative hypothesis for descent duration, peak head flexion and anxiety. Anecdotal and strong evidence for the alternative hypothesis were found for ascent duration and anxiety respectively. No differences were found in foot kinematics or margins of stability.

**Discussion:**

Simple adjustments (pinching the nose clip) to a Type IIR facemask have the benefit of reducing the lower visual field occlusion an unadjusted mask creates, and helps improve stair safety characteristics in young adults.

## 1. Introduction

Mask wearing in many parts of the world is a common practice, notably in Asian countries [1], to protect a wearer from airborne infections and/or harmful substances and is seen often in many work-related industries. Such settings include healthcare, food, construction, factories, laboratories and more. During the COVID-19 pandemic, public health experts recommended the wearing of facemasks to reduce the transmission of and exposure to COVID-19 [2–5]. Facemask wearing is now part of normal daily routines across western countries [6]. Individuals may choose to wear facemasks (particularly older adults), due to a high risk for illness, cultural reasons and/or psychological factors [7,8]. Facemasks act as a form of personal protective equipment, designed to filter the throughflow of expelled droplets [9,10] when breathing through the materials contained within the mask [11]. Type IIR surgical masks are amongst the most common types used by wearers due to their filtration efficiency [12] and loosely sit onto the wearer’s face through elastic ear loops. Guidelines from the World Health Organization recommend facemasks to fully cover the nose mouth and chin when worn, ensuring minimal gaps [5]. Type IIR surgical masks feature a nose clip which permits some contouring to the nose when pinched albeit this mask is generally considered loose fitting [13].

The wearing of facemasks, particularly those of loose fit, has been noted to occlude the lower peripheral visual field (LVF) [14–16]. The LVF provides important visual information required for safe and adaptive control of locomotion, particularly when precise stepping is required [17,18]. Facemask-related LVF occlusion raises concerns of increased fall risk particularly for older adults where the risk of falling is heightened [19] due to deteriorated vision [20,21] and/or reduced physical function [22].

The inherent risk for a stair fall and associated injury is often due to the succession of surface level changes and height from which the fall occurs [23,24]. Stair falls occur across the lifespan but are particularly common in older people. In England, from 2022 to 2023 over 39,000 falls from stairs and/or steps resulted in hospital admission of which ~35% were from children and adults below 65 years [25]. The LVF provides important information in the visuomotor processing required for safe negotiation on stairs, over raised surfaces and fall hazards [26–29], through online movement control of the leading limb [30]. Visual sampling of the stair environment for the preplanning of an appropriate stair walking action occurs approximately two to three steps in advance and determines the stair walking approach a user actions [31–33]. Occluding the LVF (and thus disrupting normal visuomotor processing) can lead to cautious movement behaviours [30,34] due to the reduction in visual information available. For example, disruptions to the LVF using custom eyewear at the penultimate step during a step-down task leads to increased vertical and horizontal heel clearances [26] or increased single limb stance times [30].

Changes in stair walking characteristics in response to disruptions in vision might also be linked to greater *conscious movement processing* (CMP) (i.e., directing attention to the monitoring and conscious control of stepping movements). Previous studies show CMP influences automatic and well-learned movements, such as locomotion and balance tasks [35–37]. Increased CMP especially occurs when people perceive a greater risk of falling and become anxious. This may happen when walking or standing at the edge of a raised platform [38], but may also occur due to an occluded visual field (as people may become aware that they may not be able to identify trip hazards in their walking path). Indeed, previous studies have reported that a postural threat leads to increased conscious processing, more cautious gait, and a reduced ability to pre-plan steps [38]. Specifically, anxious older adults visually prioritise immediate areas of their walking path (e.g., one step ahead) to consciously process the ongoing step, at the expense of previewing distal areas of a walk path (feedforward visual information). This reduces the ability to preplan movements using feedforward vision [38]. Such disruptions to visual control of gait are thought to negatively affect stepping accuracy and the risk of tripping [39,40].

Occlusion to the LVF from facemask wearing may interfere in the role lower vision provides in the preplanning of safe movement. Particularly during stair descent, the downward step level changes may occupy more of the LVF, meaning important visual information about the steps may be unavailable to the stair user if LVF is occluded. LVF occlusion might therefore lead to greater anxiety and CMP – and thus cautious stair walking behaviour. Pinching the nose clip on Type IIR facemasks may help contour the mask to the nose, reducing LVF occlusion and thereby lowering the need for cautious stair behaviour. Previous authors have commented that face masks may increase fall risk [14–16]. During overground walking, [41] found worn facemasks cause older adults to compensate through increased head flexion upon approach to (two steps prior), during and after (one step) stepping into a hole when compared to no face mask. No changes were found in gait control (speed, margins of stability, foot landing position) or lower limb electromyography activation/co-activation (medial gastrocnemius, soleus and tibialis anterior). However, to our knowledge, no study has empirically explored how facemask wearing may affect stair walking behaviour.

### 1.1. Aim

This study aimed to explore the effect of wearing a Type IIR facemask and nose clip pinch adjustment on lower visual field occlusion and stair walking behaviour. We hypothesised that LVF occlusion from an unadjusted Type IIR surgical facemask would increase anxiety and CMP and lead to increases in cautious movement behaviours. We further hypothesised that a nose clip pinch adjustment would reduce LVF occlusion, CMP and associated cautious gait behaviours. Here, we characterise cautious movement behaviour as decreases in stair walking speed, increases in foot to step edge clearance, margins of stability and foot contact on steps based on previous findings under challenging stair conditions [29,42,43].

## 2. Method

### 2.1. Participants

Eight young adults (Mean (±1SD), age: 25 [4] years, height: 1.76 (0.07) m, mass: 76 [15] kg, visual acuity: -0.3 (0.0) LogMAR, contrast sensitivity: 1.92 (0.16) LogCS; 2 females) provided written informed consent to take part in this study. All participants were free from any physical, visual or neurological impairment that would affect their normal stair walking characteristics. The Freiburg Visual test (visual acuity and contrast sensitivity) [44] was used as screening criteria for participant inclusion. Participants were excluded if scores were higher than 0.5 LogMar [45] for visual acuity, and lower than 1.5 LogCS [46] for contrast sensitivity. This investigation received institutional ethical approval.

### 2.2. Experimental design

In the following order, participants were asked to complete a LVF occlusion test (details below under Bespoke lower visual field occlusion Test) followed by stair walking (ascent and descent) trials in a single two-hour session. Each task was completed under three different conditions, presented in a randomised order: 1) no facemask worn, 2) facemask worn without any adjustments (unadjusted), 3) facemask worn with nose clip pinched by the participant to optimise the fit (customised). For the facemask conditions, participants wore Type IIR certified surgical facemasks over the nose, mouth and chin as per World Health Organisation guidelines [5]. Facemasks were secured to the head by placing the left and right ear loops around the respective ear, with the fluid repellent layer facing away from the wearer and nose wiring oriented at the top. Once secured, the facemask pleats were unfolded to ensure full covering over the nose, mouth, and chin. To adjust the fit (customised condition only), participants were asked to pinch/contour the nose clip to the bridge of the nose and twist the ear loops once (such that the loop appears as a figure of eight from a lateral view) before placing around the ears. Once secured participants were asked not to touch the facemask during testing.

### 2.3. Bespoke lower visual field occlusion test

**Fig 1:**
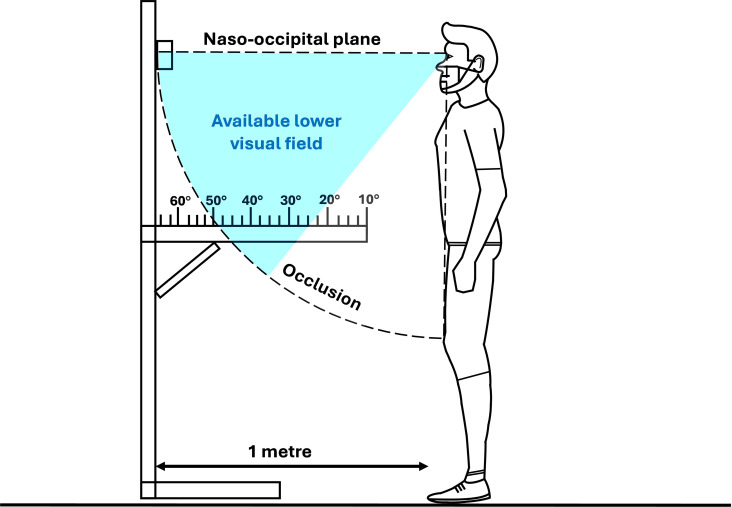
Schematic of the bespoke LVF_standing_ occlusion test performed under each condition. Each marking on the perimeter corresponds to a specific degree of visual angle within the participant’s field of view. Visible markings are within the available field of view, while non-visible markings are outside it.

We tested the extent of standing LVF occlusion (LVF_standing_) induced by mask wearing using a custom-built perimeter device (Fig 1). For the test, participants stood still and upright with their heads in a neutral position, ensuring the naso-occipital plane was perpendicular to the vertical. The perimeter device was positioned at eye level and at a viewing distance of one meter. Participants were directed by the tester to shift their gaze downwards, without flexing their heads, and identify the closest visible marking on the perimeter to themselves. Participants were asked to perform the test twice, albeit the values did not differ between measurements for every participant. The same researcher collected this data for every participant and condition. This setup thus enabled us to determine the precise degree of visual occlusion attributable to the mask worn.

### 2.4. Stair walking

For each trial, participants began approximately two to three steps away from the staircase representing the distance at which feedforward visual information about the stair environment is typically obtained [31–33]. For both stair ascent and descent, participants were asked to cross the first stair step with the same foot for each trial and walk in a step-over-step manner, continuing to the end of the landing after crossing the final step. All participants completed the stair trials at a self-selected speed and without the aid of a handrail. Each condition was block-randomised per participant and consisted of five successful stair ascent and stair descent trials performed consecutively. A seven-step custom-built staircase with step rise and going of 20 cm and 25 cm respectively [42] was used, which are within UK stair building regulations [47]. Participants wore tight clothing, flat footwear and were familiarised with the protocol prior to the recording of data. For all stair walking trials, participants were secured into an overhead fall safety harness operated by a trained belayer positioned adjacent to the staircase. Rest periods between conditions were offered to the participants throughout.

Stair walking kinematics were measured using a 26-camera motion capture system (Vicon MX, Oxford Metrics, UK) at 120 Hz. The Plug-in Gait marker set was used to model head, trunk, pelvis and lower limb kinematics with additional markers and clusters placed on the head and lower limbs for redundancy. A static calibration (anatomical pose) was captured to acquire whole body marker coordinates. A digitising wand (C-Motion, Germantown, MD, USA) was used to create virtual landmarks on the toe and heel-tips of participants’ footwear in a separate capture. Toe-tip landmarks were created on the most anterior, inferior aspect of the footwear, heel-tip landmarks were created at the most posterior, inferior aspect of the footwear. The digitising wand was also used to create virtual landmarks defining the location of all seven step edges.

### 2.5. Self-Reported outcomes

Following the stair walking task within each condition, participants were asked to complete questions designed to assess worry and somatic related anxiety. These questions have been used in previous studies assessing anxiety [43,48,49] and are originally adapted from the Sport-Anxiety Scale [50]. For worry-related anxiety, participants were asked “how worried were you when descending/ascending the stairs”. For somatic related anxiety, participants were asked “how physically anxious did you feel when descending/ascending the stairs”. Responses were averaged to give an overall indication of anxiety [49].

To assess the role of CMP, participants were asked to complete a shortened version (4-item) of the Movement Specific Reinvestment Scale [51,52], following the stair walking task within each condition. The Movement Specific Reinvestment Scale contains two 2-item subscales measuring conscious motor processing and movement self-consciousness. For conscious motor processing the items were as follows: 1. I am always trying to think about my movements when I am doing this task. 2. I am aware of the way my body moves when I am doing this task. For movement self-consciousness the items were as follows: 1. I am self-conscious about the way I look when I am doing this task. 2. I am concerned about my style of moving when I am doing this task. Responses were measured on a six-point Likert scale for each item (1 = strongly disagree; 6 = strongly agree) [52].

### 2.6 Data processing & analysis

All marker data were labelled and gap filled (quintic spline method with maximum gap of 12 frames) in Vicon (Vicon Nexus 2.6, Oxford Metrics, UK), then exported as c3d files for analysis using Visual 3D (C-Motion, Germantown, MD, USA). A fourth order Butterworth bidirectional filter (cut-off frequency 6 Hz) was used to filter the marker data. Kinematic measures included lead limb foot clearance, foot placement, margins of stability (MoS) in the anteroposterior (A/P) and mediolateral (M/L) directions, peak head flexion angle, stair LVF (LVF_stair_) occlusion and stair-walking duration.

**Fig 2:**
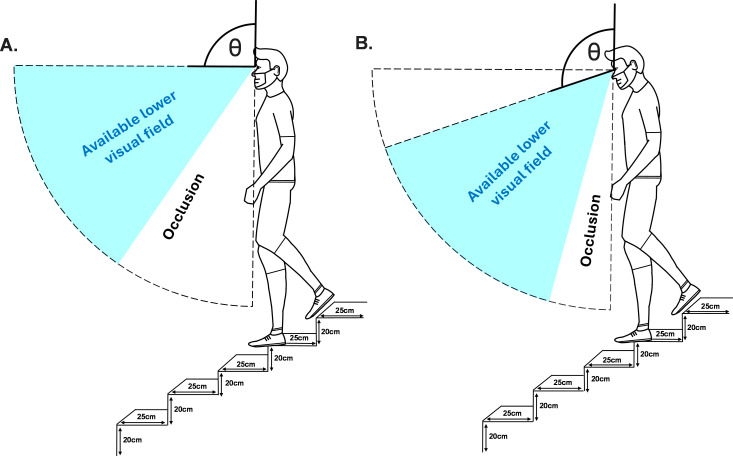
Illustrative example of stair descent occlusion calculated through subtracting the mean head flexion angle (during stair walking) from the visual occlusion (during the LVF_standing_ occlusion test). **(A)** Illustrates stair occlusion without mean head flexion angle. **(B)** Illustrates stair occlusion with mean head flexion angle whereby more of the available visual field is directed inferiorly within the sagittal quadrant (dotted lines).

Lead limb foot clearance, defined as the vertical (ascent) and horizontal (descent) distance of the virtual toe/heel tip landmark to the step edge was extracted at the point of crossing for each step edge. Foot placement was measured as the percentage of the foot in contact with the step tread distance between the virtual toe (ascent)/heel (descent) tip landmark and the virtual step edge location(s) and was extracted at the point the trail limb crossed the step edge. Head flexion was calculated as the relative joint angle formed between the head (segment) and thorax (reference segment). LVF_stair_ occlusion was calculated by subtracting the mean head flexion angle calculated during the stair negotiation from the visual occlusion measured from the bespoke LVF_standing_ occlusion test (Fig 2).

Stair walking duration was defined as the time taken from the lead limb crossing the first step edge to initial contact of the trail limb on the landing and was calculated for both ascent (stair duration_ascent_) and descent (stair duration_descent_). For calculating MoS, centre of mass (CoM) was generated as a link model-based item in Visual 3D. MoS were subsequently calculated and defined in the A/P direction as the distance between the extrapolated CoM (xCoM) and the virtual toe tip landmark (boundary of support) and in the M/L direction as the distance between the xCoM and 5th Metatarsal head (boundary of support). For stair descent trials where the virtual toe marker position was anterior to the step edge at touchdown, the step edge was used as the anterior boundary of support. In the A/P direction a negative MoS value represents an xCoM anterior to the boundary of support indicating instability. In the mediolateral direction a negative value represents an xCoM that is lateral to the boundary of support indicating instability.

xCoM was defined as:


$$xCoM=pCoM+\frac{vCoM}{\sqrt{\left({gl}^{-1}\right)}}$$

where pCoM is the A/P and M/L position of the CoM, vCoM is the instantaneous A/P and M/L velocity of the CoM, g is acceleration due to gravity, and l is the absolute distance between the CoM and the ankle joint centre [53,54]. The A/P and M/L MoS were calculated at the point of lead limb touchdown on each step.

The above variables were calculated for each participant as they represent the risk of tripping (foot clearance), slipping (foot placement) and losing balance on stairs (MoS).

### 2.7. Statistical analysis

All data were firstly checked for normal distribution through a Shapiro-Wilk test. For each kinematic variable, the average of all steps was used for statistical comparisons. A repeated measures ANOVA (α = 0.05) was used to compare the effect of each condition on all kinematic variables, self-reported measures of Conscious Movement Processing, anxiety, LVF_stair_ and LVF_standing_ occlusion. Data sphericity was assessed using Mauchly’s test of Sphericity. When data violated sphericity, a Greenhouse-Geisser (<0.75) or Huynh-Feldt (>0.75) epsilon correction was used. Where a significant main effect was found, Bonferroni post-hoc comparisons were performed to identify where the differences lie. Where data were not normally distributed a Friedman test was used. Where a significant main effect was found, a Wilcoxon signed-rank test was used to identify where the differences lie (Bonferroni corrected to α = 0.017 to account for multiple comparisons). Frequentists statistical tests were performed using SPSS 28 (SPSS version 28.0 IBM Corp, 2022). Where post hoc comparisons failed to reach statistical significance, Bayes Factors were additionally computed to indicate the evidence strength of the main effect through a Bayesian repeated measures ANOVA using default priors. Lee and Wagenmakers classification scheme indicating levels of evidence was used for Bayes Factor interpretation [55]. Levels of evidence include anecdotal (<3), moderate (>3], strong (>10], very strong (>30] and extreme (>100). Bayesian statistical tests were performed using JASP (JASP Team (2024). JASP (Version 0.19.0) [Computer software]).

## 3. Results

### 3.1. Standing lower visual field occlusion

**Fig 3:**
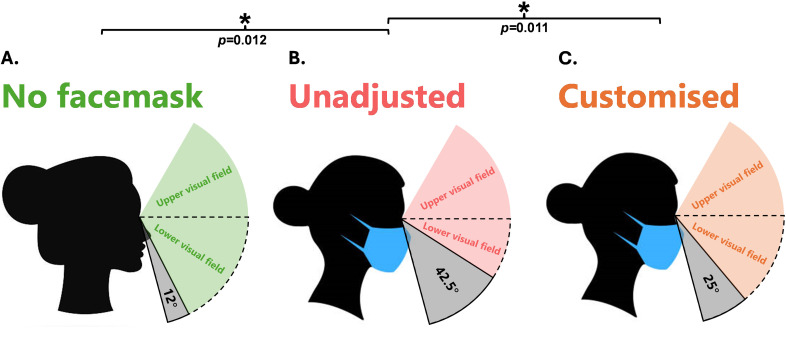
LVF_standing_ occlusion under each facemask condition. **Grey areas represent lower visual occlusion.** A = No facemask condition causing 12° of lower visual occlusion. B = Unadjusted facemask condition causing 42.5° of lower visual occlusion. C = customised facemask condition causing 25° of lower visual occlusion. * = Significant difference in post hoc comparisons between bracketed conditions.

A Friedman ANOVA test showed a significant main effect in LVF_standing_ occlusion (χ^2^(2) = 15.548, p < 0.001). Wilcoxon signed-rank post hoc comparisons showed significantly greater LVF_standing_ occlusion in the facemask unadjusted condition (median = 42.5°) compared to the no facemask condition (median = 12°) (Z = -2.521, p = 0.012). When compared to the unadjusted condition, the customised fit (median = 25°) led to significantly less LVF_standing_ occlusion (Z = -2.552, p = 0.011). No differences in LVF_standing_ occlusion were found between the no facemask condition and customised fit (Z = -2.371, p = 0.018). Fig 3 illustrates the LVF_standing_ occlusion under each condition.

### 3.2. Stair descent

#### 3.2.1. Stair lower visual field occlusion.

**Fig 4:**
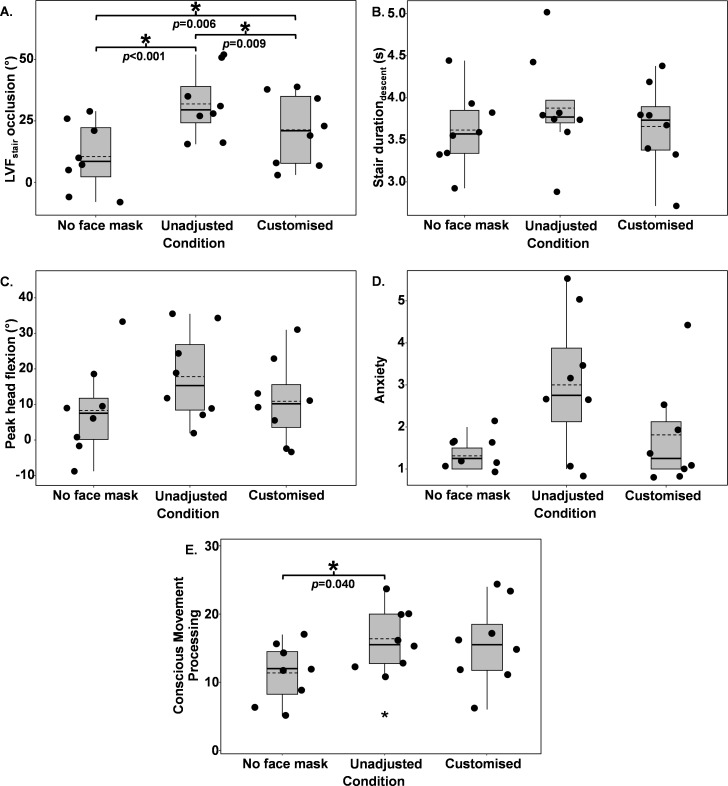
Stair descent data presented across all conditions are as follows (A), LVFstair occlusion, (B), stair duration_descent_, (C), peak head flexion, (D), anxiety and (E), Conscious Movement Processing. * = Significant difference in post hoc comparisons between bracketed conditions. --- = means within each condition. ▬ = medians within each condition.

A repeated measures ANOVA showed a significant main effect of facemask condition on descent LVF_stair_ occlusion (F(2, 14) = 43.363, p < 0.001, n2p = 0.861). Bonferroni post hoc comparisons showed the unadjusted facemask condition significantly increased descent LVF_stair_ occlusion (32° ± 14°) when compared to the no facemask (11° ± 14°, p < 0.001) and customised condition (21° ± 15°, p = 0.009). The customised condition also significantly increased descent LVF_stair_ occlusion when compared to the no facemask condition (p = 0.006) (Fig 4A).

#### 3.2.2. Stair descent duration.

A repeated measures ANOVA showed a significant main effect of facemask condition on stair duration_descent_ (F(2, 14) = 6.081, p = 0.013, n2p = 0.465). However, Bonferroni post hoc comparisons did not significantly differ between conditions (unadjusted = 3.9s ± 0.6s vs no facemask = 3.6s ± 0.5s, p = 0.055), (unadjusted vs customised = 3.7s ± 0.5s, p = 0.080), (no facemask vs customised, p = 1.000) (Fig 4B). Bayes factor analysis showed the evidence strength of the main effect in stair duration_descent_ to be moderate and 4.16 times more likely under the alternative hypothesis (increased stair duration_descent_) than the null (BF_01_ = 0.24).

#### 3.2.3. Peak head flexion.

A repeated measures ANOVA showed a significant main effect of facemask condition on peak head flexion (F(1.159, 8.113) = 8.306, p = 0.018, n2p = 0.543). However, Bonferroni post hoc comparisons did not significantly differ between conditions (unadjusted = 18° ± 13° vs no facemask = 8° ± 13°, p = 0.065), (unadjusted vs customised = 11° ± 12°, p = 0.052), (no facemask vs customised, p = 0.333), (Fig 4C). Bayes factor analysis showed the evidence strength of the main effect in peak head flexion to be moderate and 8.9 times more likely under the alternative hypothesis (increased peak head flexion) than the null (BF_01_ = 0.112).

#### 3.2.4. Self-reported outcomes.

A Friedman test showed a significant main effect of facemask condition on anxiety (χ^2^(2) = 7.000, p = 0.030). However, Wilcoxon signed-rank post hoc comparisons did not significantly differ between conditions (unadjusted = 3 ± 1.6 vs no facemask = 1.3 ± 0.4, Z = -2.207 p = 0.027), (unadjusted vs customised = 1.8 ± 1.2, Z = -1.687 p = 0.092), (no facemask vs customised, Z = -0.850, p = 0.395), (Fig 4D). Bayes factor analysis showed the evidence strength of the main effect in anxiety to be moderate and 5.9 times more likely under the alternative hypothesis (increased anxiety) than the null (BF_01_ = 0.167).

A repeated measures ANOVA showed a significant main effect of facemask condition on Conscious Movement Processing (F(2, 14) = 5.735, p = 0.015, n2p = 0.450). Bonferroni post hoc comparisons showed the unadjusted facemask condition significantly increased Conscious Movement Processing (16 ± 5) when compared to the no facemask condition (11 ± 4, p = 0.040). No differences were found between the unadjusted facemask and customised facemask condition (16 ± 6, p = 0.999) or between the no facemask and customised facemask condition (p = 0.229) (Fig 4E).

#### 3.2.5. Margins of stability and foot kinematics.

A repeated measures ANOVA showed no significant main effect of facemask condition for A/P MoS (F(2, 14) = 1.223, p = 0.324, n2p = 0.149), M/L MoS (F(2, 14) = 0.414, p = 0.669, n2p = 0.056), horizontal foot clearance (F(1.200, 8.399) = 2.284, p = 0.167, n2p = 0.246) and foot placement (F(2, 14) = 1.155, p = 0.858, n2p = 0.022). Table 1 shows mean (±1SD) for the aforementioned variables.

**Table 1:** Foot kinematics and MoS across each facemask condition. MoS are represented in the A/P and M/L directions. Values are represented as mean (±1SD). Negative and positive MoS values represent instability and stability respectively. Foot placement represents the percentage of foot in contact with the step tread.

Stair descent			
	No facemask	Facemask unadjusted	Facemask customised
**Horizontal foot clearance (m)**	0.084 (0.022)	0.088 (0.025)	0.083 (0.021)
**Foot placement (%)**	82.7 (8.3)	82.4 (8.1)	82.6 (7.7)
**MoS A/P (m)**	-0.031 (0.037)	-0.044 (0.044)	-0.035 (0.052)
**MoS M/L (m)**	0.042 (0.021)	0.042 (0.024)	0.037 (0.025)
**Stair ascent**			
	**No facemask**	**Facemask unadjusted**	**Facemask customised**
**Vertical foot clearance (m)**	0.050 (0.016)	0.056 (0.016)	0.053 (0.014)
**Foot placement (%)**	73.5 (9.2)	75.4 (11.8)	74.6 (8.6)
**MoS A/P (m)**	0.093 (0.037)	0.107 (0.043)	0.099 (0.045)
**MoS M/L (m)**	0.128 (0.018)	0.131 (0.020)	0.129 (0.019)

### 3.3. Stair ascent

#### 3.3.1. Stair lower visual field occlusion.

**Fig 5:**
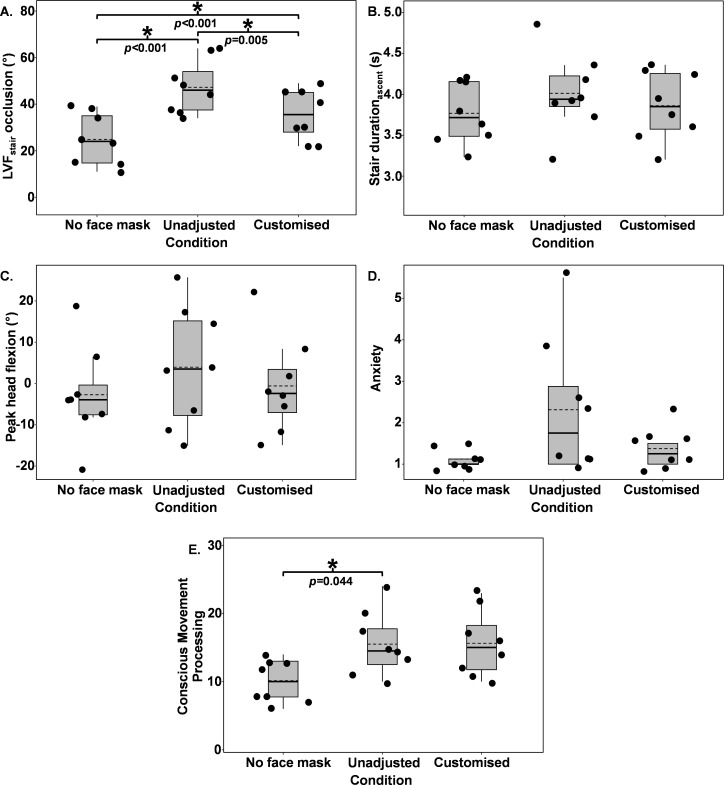
Stair ascent data presented across all conditions are as follows (A), LVFstair occlusion, (B), stair duration_ascent_, (C), peak head flexion, (D), anxiety and (E), Conscious Movement Processing. * = Significant difference in post hoc comparisons between bracketed conditions. --- = means within each condition. ▬ = medians within each condition.

A repeated measures ANOVA showed a significant main effect of facemask condition on ascent LVF_stair_ occlusion (F(2, 14) = 62.331, p < 0.001, n2p = 0.899). The unadjusted facemask condition significantly increased ascent LVF_stair_ occlusion (47° ± 12°) when compared to the no facemask (25° ± 11°, p < 0.001) and customised condition (35° ± 11°, p = 0.005). The customised condition also significantly increased ascent LVF_stair_ occlusion compared to the no facemask condition (p < 0.001) (Fig 5A).

#### 3.3.2. Stair duration.

A repeated measures ANOVA showed a significant main effect of facemask condition on stair duration_ascent_ (F(2, 14) = 4.785, p = 0.026, n2p = 0.406). However, Bonferroni post hoc comparisons did not significantly differ between conditions (unadjusted = 4.0s ± 0.5s vs no facemask = 3.8s ± 0.4s, p = 0.105), (unadjusted vs customised = 3.9s ± 0.4s, p = 0.397), (no facemask vs customised, p = 0.294) (Fig 5B). Bayes factor analysis showed the evidence strength of the main effect in stair duration_ascent_ to be anecdotal and 2.46 times more likely under the alternative hypothesis (increased stair duration_ascent_) than the null (BF_01_ = 0.406).

#### 3.3.3. Peak head flexion.

A repeated measures ANOVA showed no significant main effect of facemask condition on peak head flexion during stair ascent F(2, 14) = 3.002, p = 0.082, n2p = 0.300), (unadjusted = 4° ± 14°, no facemask = -3° ± 12°, customised = -1° ± 12°), (Fig 5C).

#### 3.3.4. Self-reported outcomes.

A Friedman test showed a significant main effect of facemask condition on anxiety during stair ascent (χ^2^(2) = 7.600, p = 0.022). However, Wilcoxon signed-rank post hoc comparisons did not significantly differ between conditions (unadjusted = 2.3 ± 1.7 vs no facemask = 1.1 ± 0.2, Z = -1.826 p = 0.068), (unadjusted vs customised = 1.4 ± 0.5, Z = -1.841, p = 0.066), (no facemask vs customised, Z = -1.633, p = 0.102). Bayes factor analysis showed the evidence strength of the main effect in stair ascent anxiety to be strong and 17.69 times more likely under the alternative hypothesis (increased anxiety) than the null (BF_01_ = 0.057).

A repeated measures ANOVA showed a significant main effect of facemask condition on stair ascent Conscious Movement Processing (F(2, 14) = 8.566, p = 0.004, n2p = 0.550). Bonferroni post hoc comparisons showed the unadjusted facemask condition significantly increased stair ascent Conscious Movement Processing (16 ± 5) when compared to no facemask (10 ± 3, p = 0.044). Bonferroni post hoc comparisons showed no further main effects between conditions (unadjusted vs customised = 16 ± 5, p = 0.999), (no facemask vs customised, p = 0.075) (Fig 5E).

#### 3.3.5. Margins of stability and foot kinematics.

Table 1 shows mean (±1SD) data for vertical foot clearance, foot placement, and MoS during stair ascent. A repeated measures ANOVA showed no significant main effect of facemask condition for stair ascent vertical foot clearance F(2, 14) = 3.380, p = 0.063, n2p = 0.326), foot placement F(2, 14) = 0.425, p = 0.662, n2p = 0.057), A/P MoS F(2, 14) = 2.298, p = 0.137, n2p = 0.247), or M/L MoS F(2, 14) = 1.536, p = 0.249, n2p = 0.180).

## 4. Discussion

To the authors knowledge, we present the first study to explore how the wearing and fit of Type IIR facemasks affect stair safety in a young adult sample. Overall, we show that an unadjusted Type IIR facemask leads to significant increases in occlusion during LVF_standing_ and LVF_stair_, which can be reduced through adjusting the facemask (pinching the nose clip). Unadjusted facemasks increase Conscious Movement Processing, and mainly with moderate evidence, increases stair duration, peak head flexion and anxiety. Foot kinematics and MoS remain unaffected despite the benefit of reduced LVF_stair_ occlusion when the facemask is adjusted or when no facemask is worn.

### 4.1. Stair lower visual field occlusion

During stair descent, the steps progress towards the ground landing meaning a significant proportion of the stairs may fall within the LVF and field of occlusion. Greater peak head flexion values during descent compared to ascent likely represents behaviour for viewing the steps in the available/unaffected LVF for visuomotor planning [29,52]. Our findings indicate a visual benefit when pinching the nose clip on a Type IIR facemask in reducing some of the occluded LVF (~17.5°). This may be particularly useful when negotiating lowly lit, uniform or visually ambiguous stairs (sub optimal conditions) where increased reliance on vision (and the LVF) may be expected for online control of the lead limb [17,56], detection of step edges [57,58] and judgment of step dimensions [59,60].

### 4.2. Peak head flexion

The lack of strong evidence for unadjusted facemasks increasing stair descent peak head flexion, despite significant increases in LVF_stair_ from an unadjusted facemask, suggests the amount of head flexion observed was enough for participants to complete the stair descent. Additional increases in peak head flexion to restore all the LVF_stair_ might negatively affect the ability for feedforward visual planning, the subsequent stepping action [61], and the role of peripheral vision in balance [62]. This could explain the absence of strong evidence of differences between conditions in peak head flexion and why participants were still able to safely descend stairs. Consistent foot kinematics and MoS across the facemask conditions seem to further support this. The absence of any peak head flexion effect during stair ascent may also relate to a larger proportion of the stairs falling within the higher/unaffected field of vision and/or the greater fall threat associated with stair descent compared to ascent [63]. It may have also been possible that our participants relied more on somatosensory feedback of the step dimensions to guide their stepping behaviour, however this would need to be explicitly tested.

### 4.3. Movement behaviours

Compared to previous young adult stair studies, Graci, and colleagues [64] reported increases in vertical forefoot clearance over the first and second step (five step staircase) under LVF occlusion in young adults and Miyasike-daSilva and colleagues [29], found increases in stair walking time under lower visual field occlusion in young adults. Here, we show moderate (stair duration_descent_) to anecdotal (stair duration_ascent_) evidence of increased stair duration and no main effect on foot kinematics or MoS. Differences in findings to the cited studies might relate to the degrees of occlusion and previous experiences wearing facemasks. Graci, and colleagues [64] indicate their participants needed 90° of head flexion to view the upcoming step and/or their feet. In comparison, we report visual occlusions here of ~32° (standing occlusion = 42.5°), suggesting larger degrees of LVF occlusion were used by Graci, and colleagues. Miyasike-daSilva and colleagues [29] used safety goggles with adhesive paper to occlude the LVF. Facemasks were routinely worn during the pandemic through mandatory regulation and are worn as standard in many industries which might have compounded any acute exposure effects occlusion from facemasks may have.

Stronger differences in stair behaviours may be likelier in older adults due to natural age-related deterioration and consequences of a stair fall [23,63]. To our knowledge, studies have not assessed how LVF occlusion from facemasks affect older adult stair safety but have assessed the LVF occlusion effect during overground walking/obstacle crossing. Recent findings show wearing a facemask causes older adults to increase head flexion upon approach to (two steps prior), during and after (one step) stepping into a hole during overground walking [41]. No changes were found in gait control (speed, margins of stability, foot landing position) or lower limb electromyography activation/co-activation (medial gastrocnemius, soleus and tibialis anterior). Under LVF occlusion, other studies show increased head flexion and reduced gait speed during overground walking in older adults on different terrain [56]. Findings by [65] show no changes in older adult walking speed and postural stability during obstacle crossing. The present study partially confirm previous studies as similar to [41] and [65], we found locomotive behaviour to be largely unchanged, however we did not find strong/significant differences in peak head flexion. Other differences to the previous studies might be linked to the locomotive task under the visual conditions, thus future investigations should examine how facemasks affect older adult stair walking behaviour.

### 4.4. Conscious movement processing & anxiety

The LVF occlusion from an unadjusted facemask significantly increased CMP (ascent and descent) when compared to no facemask. The sensory information associated with facemask wearing (i.e., how the mask is positioned/sits on the face and/or altered respiration) might also have contributed to the increases in conscious processes. Increases in CMP have been linked to a reduced ability to utilise vision in a feedforward manner as individuals attend internally towards movement and less towards the visual environment [38]. On stairs, this may translate to a reduced ability to look at the steps ahead for the visual planning of an appropriate and safe stair negotiation action, with increased attention directed towards the ongoing step. Increases in CMP reportedly lead to decreased postural stability, increased gait variability [66] and increased stepping errors in older adults [61]. Prioritisation of immediate visual information has been suggested as a requirement to consciously control each individual step and has been associated with heightened fall-related anxiety [38]. Here we observed moderate (descent) to strong (ascent) evidence for increased anxiety but found no accompanying differences in MoS or peak head flexion between conditions. Elevated anxiety and CMP may more readily result in changes in (visual control of) gait in older adults compared to healthy young adults that were part of our study..

## 5. Limitations

A limitation of the presented study is the absence of gaze behaviour. Despite our measures of peak head flexion, we could not directly evidence the visual allocation to immediate steps on stairs under heightened Conscious Movement Processing and account for the role of saccades in the collection of stair visual information. Whilst this study is exploratory, we acknowledge the small sample hinders the ability to determine where differences do or don’t exist between each experimental condition. A larger sample size and inclusion of older adults would have been favourable. However, we supplement the presence of main effects with Bayesian inference to indicate the strength of evidence and present a robust and rigorous approach to the statistical analyses.

## 6. Conclusion

Overall, our exploratory results show adjustments (pinched nose clip) to a Type IIR facemask have the benefit of reducing the LVF occlusion an unadjusted mask creates in young adults. The effect of LVF occlusion from an unadjusted facemask increases Conscious Movement Processing, and mainly with moderate evidence, increases stair duration, peak head flexion and anxiety during stair negotiation. Foot kinematics and dynamic stability however remains unaffected. Future research should determine how the facemask conditions affect older adult stair behaviour, where stronger differences may be found due to the heightened stair fall risk. [23,25,67,68].

## Supporting information

S1 Data Results of standing lower visual field occlusion, stair descent and stair ascent outome variables for each participant.
(XLSX)
